# The Distribution of *Bathyarchaeota* in Surface Sediments of the Pearl River Estuary Along Salinity Gradient

**DOI:** 10.3389/fmicb.2020.00285

**Published:** 2020-02-27

**Authors:** Dayu Zou, Jie Pan, Zongbao Liu, Chuanlun Zhang, Hongbin Liu, Meng Li

**Affiliations:** ^1^SZU-HKUST Joint Ph.D. Program in Marine Environmental Science, Institute for Advanced Study, Shenzhen University, Shenzhen, China; ^2^Department of Ocean Science, The Hong Kong University of Science and Technology, Hong Kong, China; ^3^Shenzhen Key Laboratory of Marine Microbiome Engineering, Institute for Advanced Study, Shenzhen University, Shenzhen, China; ^4^Shenzhen Key Laboratory of Marine Archaea Geo-Omics, Southern University of Science and Technology, Shenzhen, China

**Keywords:** archaeal community structure, *Bathyarchaeota*, *Thermoprofundales*, surface sediment, Pearl River estuary

## Abstract

*Bathyarchaeota*, a recently proposed archaeal phylum, is globally distributed and highly abundant in anoxic sediments. Metabolic pathways of the *Bathyarchaeota* members are diverse and, hence, this phylum has been proposed to play an important role in global biogeochemical cycles. *Bathyarchaeota* members are distributed in the estuarine environments. However, limited information is available about their detailed community structure, abundance, and functions in the Pearl River estuary (PRE). In the current study, we performed a comprehensive investigation of the archaeal community in the PRE surface sediments along a salinity gradient, with a focus on *Bathyarchaeota*. *Bathyarchaeota* was the dominant archaeal phylum, with the abundance of the bathyarchaeotal 16S rRNA gene ranging from 1.43 × 10^8^ to 1.22 × 10^9^ copies/g sediment dry weight (d.w.), and Bathy-8 was the dominant subgroup. *Thaumarchaeota*, *Lokiarchaeota*, and *Euryarchaeota*, including *Thermoprofundales* (MBG-D archaea), were the other major archaeal groups in the PRE. The differences of community distributions in the high- and low-salinity sediments were hence investigated. Statistical analysis revealed that besides salinity, ammonium, and total organic carbon were the most important environmental factors influencing the archaea community structure, including that of *Bathyarchaeota*, in the PRE. The archaeal network indicated the cooccurrence among *Bathyarchaeota*, *Lokiarchaeota*, and *Euryarchaeota*, while Bathy-6 presented unique correlations compared with other bathyarchaeotal subgroups. These observations indicate that *Bathyarchaeota* may play a role in ecosystem function through microbe–microbe interactions, revealing a possible different lifestyle for Bathy-6 in eutrophic estuarine sediments.

## Introduction

Marine sediments serve as an immense reservoir of carbon sources that support the formation of unique ecosystems of numerous benthic organisms. A previous study revealed that approximately 2.9 × 10^29^ microbial cells reside in marine sediments, which is similar to the cellular abundance in seawater (Kallmeyer et al., 2012). Without a doubt, sedimentary microbes play major roles not only in benthic environments but also in global geochemical cycles (Fry et al., 2008), because they are capable of degrading diverse organic compounds mainly deposited from water column and land (Berner, 1982; Wollast, 1991; Sundby et al., 1993).

Estuaries act as connectors between land and ocean, and, hence, exhibit unique characteristics that are different from those of terrestrial and oceanic environments (McLusky et al., 2004). The estuarine ecosystems are particularly affected by the large amount of suspended sediment carried by a river discharge (Milliman and Meade, 1983). The Pearl River is located in the southern part of China. It delivers approximately 8.5 × 10^7^ tons of sediments into the South China Sea (SCS) every year, and accounts for over 80% deposition of the suspended particulate matter in the Pearl River estuary (PRE) (Zhou et al., 2004). Because of the industrialization and urbanization along the Pearl River Delta area, massive terrigenous wastes have been released into the estuary, resulting in a severe contamination of the aquatic and benthic environments (Huang et al., 2003). Although undoubtedly the microbes play irreplaceable roles degrading the polluting chemicals, investigations on ecological functions of the majority of sedimentary microbes in the eutrophic PRE were limited.

Archaea are an indispensable component of the sedimentary ecosystems, and the abundance of *Archaea* is similar to that of *Bacteria* in many regions (Danovaro et al., 2015; Hoshino and Inagaki, 2018; Flemming and Wuertz, 2019). According to a recent study, 37.3% of all microbial cells in sedimentary environments are archaeal cells, with the proportion varying between 40 and 12.8% in the ocean margin and open-ocean sites, respectively (Hoshino and Inagaki, 2018). *Bathyarchaeota* [formerly called Miscellaneous Crenarchaeotal Group (MCG)], a representative sedimentary archaeal phylum, is widely distributed in various environments, such as marine and terrestrial sediments, hot springs, hydrothermal vents, etc. (Inagaki et al., 2006; Kubo et al., 2012; Meng et al., 2014; Zhou et al., 2018a). Liu et al. (2018) surveyed the archaeal communities in 24 estuaries from different latitude regions, and reported that *Bathyarchaeota* dominate the estuarine sedimentary archaeal community, especially in the middle and low latitude regions. Genomic analysis uncovered the metabolic capacity of *Bathyarchaeota*, revealing that these microbes can utilize a diverse range of organic matter, such as detrital proteins, aromatic compounds, and plant-derived carbohydrates (Meng et al., 2014; Lazar et al., 2016; Zhou et al., 2018a). Intriguingly, genes related to acetogenesis and methane metabolism have been identified in the genomes of some bathyarchaeotal members (Evans et al., 2015; He et al., 2016; Lazar et al., 2016), indicating the high metabolic versatility of *Bathyarchaeota*.

Originally, at least 17 subgroups of *Bathyarchaeota* were identified based on the 16S rRNA gene similarity (Kubo et al., 2012), while a recent study has classified *Bathyarchaeota* into 25 subgroups (Zhou et al., 2018a). The distribution pattern of different subgroups of *Bathyarchaeota* is likely related to geochemical parameters, and the predominant subgroup type varies with the environment (Zhou et al., 2018a). For instance, the total organic carbon (TOC) content is directly, strongly, and positively correlated with the bathyarchaeotal 16S rRNA gene abundance in the sediment cores of the SCS, and Bathy-8 dominates in the deep sediment layers and Bathy-10 is predominant in the shallow layers (Yu et al., 2017). The abundance of bathyarchaeotal subgroups in the surface sediments of the northern SCS also correlates with the seawater depth, with Bathy-6 predominant in the shallow water sediments (Zhou et al., 2018b). Further, in the White Oak River estuary, the reductive redox conditions strongly influence the abundance and distribution of *Bathyarchaeota*, with Bathy-6 and Bathy-8 dominating in the shallow and deep sediments, respectively (Lazar et al., 2015). Although the presence of sedimentary *Bathyarchaeota* in the PRE has been previously reported (Liu et al., 2014; Xie et al., 2014), the abundance and detailed subgroup types of *Bathyarchaeota* in this region have not yet been thoroughly investigated.

In the current study, we surveyed the abundance and distribution of archaea in the surface sediments in the PRE, focusing on *Bathyarchaeota*. We aimed to: (1) determine the abundance of *Bathyarchaeota* in the PRE surface sediments along the salinity gradient from the inner estuary to the outer continental shelf; (2) identify the distribution pattern of bathyarchaeotal subgroups and other major archaea in the PRE; (3) explore the correlations between the bathyarchaeotal subgroups and physicochemical parameters, as well as other archaeal groups; and (4) understand the niche difference of the surface sedimentary *Bathyarchaeota* in the PRE.

## Materials and Methods

### Sampling and Measurements of Physicochemical Parameters

**FIGURE 1:**
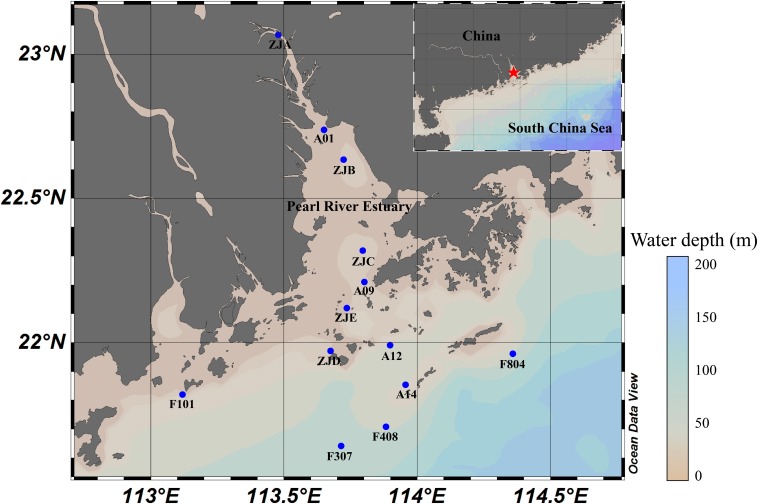
Sampling stations in the PRE. Inset, overview of the studied area. Red star, the research area; blue dots, sampling stations.

Surface sediment samples from the PRE region (21.64° to 23.07° N, and 113.71° to 114.36° E) along the salinity gradient were collected during two boat cruises in July 2015 (samples ZJA, ZJB, ZJC, ZJD, and ZJE) and July 2017 (samples A01, A09, A12, A14, F101, F307, F408, and F804) (Figure 1). The sediments were sealed in 50 ml tubes (Falcon) immediately after sampling of the water bed, stored in liquid nitrogen on board, and then transferred to a −40°C refrigerator in the laboratory for further analysis. Water samples from wet sediment samples were collected by centrifugation, and the pH and salinity were determined by using with a pH meter (FE20/EL20, Mettler Toledo) and a salinity meter (MASTER-S/MillM, Atago) on board. Sediment samples (5 g each) were air-dried to calculate the dry weight (d.w.) and to measure physicochemical parameters in the laboratory. The concentrations of ^NH_4^+^, ^NO_2^–^, and ^NO_3^–^ were measured using AutoAnalyzer 3 HR (Seal, United States) (Zhou et al., 2017). The TOC, inorganic carbon (IC), total carbon (TC) and total nitrogen (TN) content, were analyzed using TOC-L_CPH/CPN_ (Shimadzu, Japan) as reported previously (Pan et al., 2019).

### DNA Extraction, Sequencing, and Sequence Data Processing

DNA was extracted from 10 g of the wet sediment in triplicate for each sample by using the PowerMax soil kit (Qiagen) following the manufacturer’s instructions, all triplicates from each sample were combined for the subsequent sequencing and analysis, respectively. The primers Arch524F (5′-TGYCAGCCGCCGCGGTAA-3′) and Arch958R (5′-YCCGGCGTTGAVTCCAATT-3′) were employed for archaeal 16S rRNA amplification, as described in recent studies (Cerqueira et al., 2017; Cui et al., 2019; Wang et al., 2019). The amplicons were paired-end sequenced (2 bp × 250 bp) using an Illumina HiSeq 2500 platform following standard protocols. Sequence data was analyzed using the QIIME2 pipeline (version 2018.4), following the procedures for quality filtration, demultiplexing, denoising with dada2 (Callahan et al., 2016), taxonomy assignment, and phylogenetic and diversity analysis (Bolyen et al., 2018). Taxonomic assignment of the representative archaeal sequences was achieved using the SILVA database (release 132) (Quast et al., 2012). The sequences of *Bathyarchaeota* (Zhou et al., 2018a), *Thermoprofundales* [Marine Benthic Group D (MBG-D)] (Zhou et al., 2019), and *Lokiarchaeota* (Cai et al., 2019) were categorized into different subgroups based on recent studies, as appropriate. The archaeal reads were then extracted; the operational taxonomic unit (OTU) table was normalized by setting the uniform sequence number to 51,331 for each sample. Diversity analysis was performed using the command “qiime diversity core-metrics-phylogenetic” before downstream analyses. The alpha diversity of archaeal community was determined by the Shannon and Simpson indices, and the number of observed species was generated by QIIME2.

### Quantitative PCR Analysis

To quantify the 16S rRNA gene copy numbers of total archaea and *Bathyarchaeota* in each sediment sample, real-time quantitative PCR (qPCR) and analysis were done using the QuantStudio3 instrument (Thermo Fisher Scientific), and the primer pairs Arch519F (5′-CAGCCGCCGCGGTAA-3′)/ Arch908R (5′-CCC GCC AAT TCC TTT AAG TT-3′) (Ovreås et al., 1997; Jorgensen et al., 2012) and MCG242dF (5′-TDACCGGTDCGGGCCGTG-3′)/Bathy442R (5′-GGCGG CTGACACCAGTCT-3′) (Pan et al., 2019) were used respectively. For the analysis, a 20-μl qPCR reaction was prepared, containing the following: 10 μl of PowerUp SYBR Green master mix (Applied Biosystems), 2 μl of DNA template, 1 μl each of the forward and reverse primers (10 μM), and 6 μl of ddH_2_O, and standard qPCR curves were generated using sequential 10-fold dilution series of the pMD19-T vector, as previously described (Pan et al., 2019). The gene copy numbers in the standard dilution series were calculated by first measuring the DNA concentration by Nanodrop (Thermo Scientific) and then applying the equation: abundance of gene copy number/μl = (amount/μl × 6.022 × 10^23^)/(length × 1 × 10^9^ × 324.5). The properties for the final adjusted standard curve for total archaea were: *r*^2^ = 0.997, efficiency% = 86.84%, and for *Bathyarchaeota* were: *r*^2^ = 0.999, efficiency% = 97.20%.

### Statistical Analysis

Sample clustering was employed using PAleontological Statistics (PAST, version 3.16) at archaeal OTU level and the unweighted pair-group method with arithmetic means (UPGMA). Based on the clustering results, the samples were categorized into two groups, Group A (stations ZJA, A01, ZJB, ZJC, and ZJE) and Group B (stations A09, A12, A14, F101, F307, F408, F804, and ZJD) (Supplementary Figure S2). The normality of data, including those for the physicochemical parameters and diversity indices, was examined using the Kolmogorov–Smirnov test. The *t*-test was employed to evaluate the significance of differences between two groups of data with normal distribution, whereas the Mann–Whitney test was used when the data did not pass the normality test. Analysis of similarities (ANOSIM) was implemented using PAST to test the differences in the total archaeal and bathyarchaeotal community compositions in different sample groups.

Pearson correlation analysis was used to describe the correlative relationship between the environmental parameters measured in the current study and the relative abundance, quantity, and diversity index of bathyarchaeotal and total archaeal community was conducted using IBM SPSS Statistics 20. The correlation coefficient matrix was generated using bootstrap and two-tailed *p*-value statistics. Principal coordinate analysis (PCoA) was employed to delineate the dissimilar relationship between samples based on the OTU composition for the total archaeal community and bathyarchaeotal subgroups (PAST, version 3.16). Linear discriminant analysis (LDA) effect size (LEfSe) was employed for certain subgroups of the major archaeal groups (*Bathyarchaeota*, *Thaumarchaeota*, *Lokiarchaeota*, and *Euryarchaeota*) on the OTU level when the average abundance fraction was larger than 0.1%; the α-value for the factorial Kruskal–Wallis test was 0.05 and the threshold for the logarithmic LDA score was 2.0 (Segata et al., 2011). Redundancy analysis (RDA) was conducted using CANOCO (version 5.0) to explain the influence of environmental factors on the ordination of samples and the compositional archaeal taxa. Variation partitioning analysis (VPA) was employed to quantify the first three contributing factors by RDA ordination on the composition of *Bathyarchaeota* (Šmilauer and Lepš, 2014). To depict the co-occurrence of bathyarchaeotal subgroups and other major archaeal taxa, Cytoscape (version 3.7.0) and CoNet (version 1.1.1 beta) were used for network analysis on the OTU level involving three approaches (Pearson correlation, Spearman correlation, and Bray–Curtis dissimilarity). Nodes with the relative abundance over 0.1% and edges consistent with the two methods (coefficient > 0.7 or < –0.7, and *p* < 0.01) were retained (Shannon et al., 2003; Faust and Raes, 2016).

### Sequencing Data Availability

The raw HiSeq sequencing data for 13 archaeal 16S rRNA gene libraries in this study were deposited in NCBI Sequence Read Archive (SRA) database with the BioProject accession no. PRJNA574836 and the BioSample accession nos. from SAMN12869739 to SAMN12869751.

## Results

### Physicochemical Characteristics and Archaeal Diversity of Samples

The sampling location and environmental factors of each sediment sample are specified in Figure 1 and Supplementary Table S1, respectively. The water depth of the sampling sites ranged from approximately 8 m to over 40 m, while the salinity varied from 0.3 to 34.5‰ along the PRE. Among the physiochemical factors, ammonium, TOC, and TC of Group A samples were significantly higher than those of Group B samples (*p* < 0.05, Supplementary Table S2). And Group B samples had relatively higher salinity (0.3 to 26.2‰) than Group A samples (31.3 to 34.5‰) (*p* < 0.05, Supplementary Tables S1, S2). Nevertheless, the concentrations of nitrite, nitrate, and TN were not significantly different between the samples regardless of salinity (Supplementary Table S2).

Following dada2 denoising and taxonomy assignment, the archaeal reads were extracted for further analysis. The maximum archaeal read number in the 13 samples was 81,307 (at station A14) and the minimum number was 51,331 (at station F101) (Supplementary Table S3). The diversity indices for each sample, including the observed species number, and the Shannon and Simpson indices, are listed in Supplementary Table S3. The rarefaction curves of the archaeal community indicated that all samples achieved a plateau at the applied sequencing depth (Supplementary Figure S1). No significant differences of diversity index were apparent between archaeal communities in different sample groups (Supplementary Table S2).

### Community Composition and Abundance of Total Archaea and *Bathyarchaeota*

**FIGURE 2:**
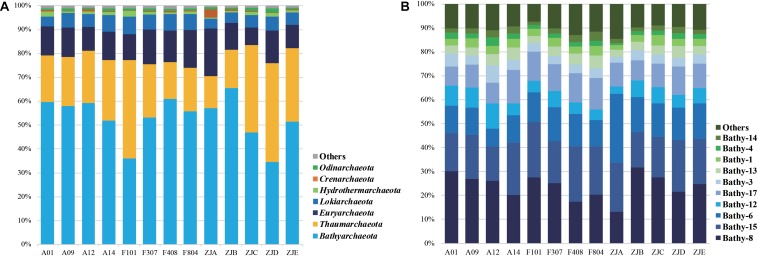
Archaeal community composition on the phylum level **(A)** and major bathyarchaeotal **(B)** subgroup composition (average abundance fraction > 1%).

The composition of archaeal community on the phylum level is shown in Figure 2A and Supplementary Table S4. *Bathyarchaeota* were dominant in almost all sediment samples, except for samples ZJD and F101, and the abundance varied from 34.6 to 65.5%. *Thaumarchaeota* ranked second, except for samples ZJD and F101, in which the relative abundance (41.4 and 41.1%, respectively) was slightly higher than that of *Bathyarchaeota* (34.6 and 36.1%, respectively). Furthermore, the average relative abundance of *Euryarchaeota* and *Lokiarchaeota* were 12.5 and 5.7%, respectively.

The composition of *Bathyarchaeota* is illustrated in Figure 2B. We observed 22 subgroups (Bathy-1, -2, -3, -4, -5a, -5b, -5bb, -6, -8, -9, -10, -11, -12, -13, -14, -15, -16, -17, -18, -19, -20, and -22) in the PRE surface sediments. Bathy-8 was the dominant subgroup, followed by Bathy-15 and Bathy-6. The community compositions of *Euryarchaeota*, *Thaumarchaeota*, and *Lokiarchaeota* were also analyzed. *Thermoprofundales* (MBG-D), *Nitrosopumilales* (Marine Group I, MG-I), and the Loki-3 subgroup represented the major groups in these communities, in addition to *Bathyarchaeota*, respectively (Supplementary Figure S3). Within the order *Thermoprofundales*, 14 subgroups were observed (MBGD-1, -2, -3, -5, -6, -8a, -8b, -8c, -9a, -9b, -9c, -10, -11, and -12). MBGD-8c was the dominant group, followed by MBGD-8a and MBGD-12 (Supplementary Figure S3B). *Methanosarcinales* dominated in the methanogen community within *Euryarchaeota*, and *Methanomicrobiales* and *Methanofastidiosales* were also observed (Supplementary Figure S3B).

**FIGURE 3:**
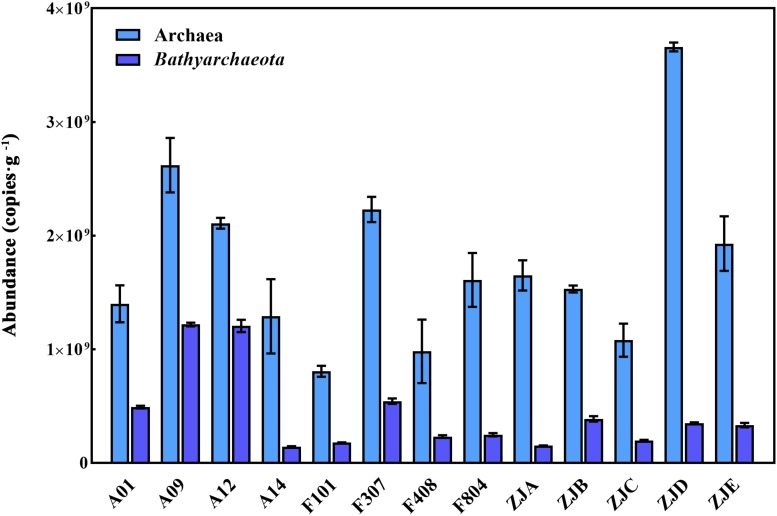
The abundance of archaeal and bathyarchaeotal 16S rRNA genes in each sample. Light blue bars, the total archaea; dark blue bars, the *Bathyarchaeota*.

The abundance of archaea and *Bathyarchaeota* was quantified using qPCR analysis of the 16S rRNA gene (Figure 3 and Supplementary Table S3). The observed total archaeal and bathyarchaeotal 16S rRNA gene abundance ranged from 8.06 × 10^8^ to 3.66 × 10^9^ and 1.43 × 10^8^ to 1.22 × 10^9^ gene copies/g sediment d.w., respectively.

### Relationship Between the Community Properties and Physicochemical Parameters

Principal coordinate analysis diagrams delineating the different community compositions of *Bathyarchaeota* and the total archaea in different sample groups are shown in Supplementary Figure S4. The first PCo explained 24.35% of the variation, while the second PCo explained 19.04% of the variation. When the bathyarchaeotal subgroup composition was analyzed on the OTU level, samples were clustered more closely for each group and the first and second PCo explained 23.06 and 22.28% of variation, respectively. In addition, PCoA results showed that there were no clear boundaries for samples from different years regarding to both total archaeal and bathyarchaeotal community.

**FIGURE 4:**
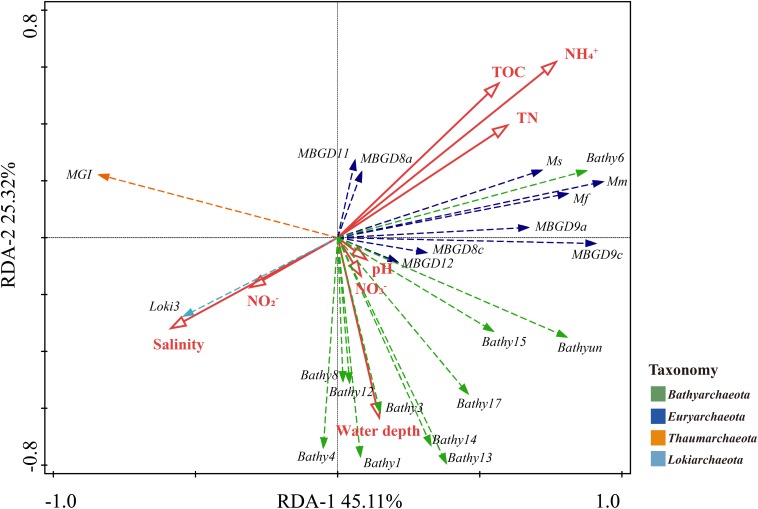
RDA results for the relationship between the physicochemical parameters and the subgroups of *Bathyarchaeota*, *Thaumarchaeota*, *Lokiarchaeota*, and *Thermoprofundales*. Solid lines, physicochemical parameters; dashed lines, archaeal subgroups. Mm, Mf, and Ms stand for *Methanomicrobiales*, *Methanofastidiosales*, and *Methanosarcinales*, respectively.

Further, RDA indicated that ammonium, salinity and TOC were the three most important contributing factors that shaped the archaeal community, explaining 32.3, 21.3, and 20.6% (*p* < 0.05) of total variation, respectively (Figure 4). The subgroups of *Thermoprofundales* and Bathy-6 were closely associated with ammonium, TOC and TN, whereas MG-I and Loki-3 were mainly affected by the salinity and nitrite. Almost all bathyarchaeotal subgroups were closely related with the water depth, except for Bathy-6, which was separate from other bathyarchaeotal subgroups.

Pearson correlation analysis was done to describe the relationship between the physicochemical parameters and variables including 16S rRNA gene abundance of archaea and *Bathyarchaeota*, the compositional proportion of archaeal phyla, and the diversity index (Supplementary Table S5). The analysis revealed that the 16S rRNA gene abundance of archaea and *Bathyarchaeota* did not show strong correlations with any of the parameters investigated or with the diversity index, suggesting an overall uniformity of *Bathyarchaeota* in the PRE. With respect to the archaeal composition, the *Lokiarchaeota* fraction was significantly negatively correlated with salinity (*p* < 0.001), and ammonium, TOC and TN (*p* < 0.05). By contrast, the *Crenarchaeota* fraction exhibited opposite trends than *Lokiarchaeota*.

**FIGURE 5:**
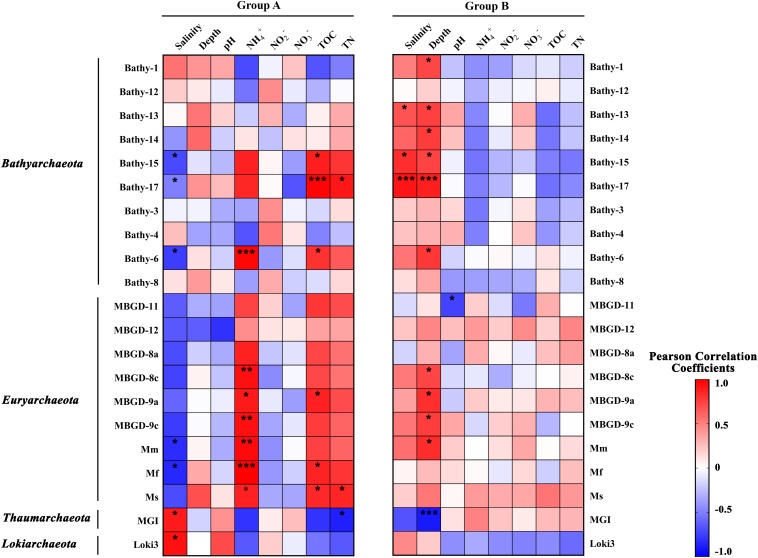
The Pearson correlation coefficients between physicochemical parameters and major archaeal subgroups in Group A (relatively low salinity) and Group B (relatively high salinity). Red squares, positive correlations; blue squares, negative correlations. *, **, and *** represent the significance at 0.05, 0.01, and 0.001 level, respectively. Mm, Mf, and Ms stand for *Methanomicrobiales*, *Methanofastidiosales*, and *Methanosarcinales*, respectively.

The results of Pearson correlation analysis on major archaeal subgroups in sample groups was illustrated in Figure 5. In the relatively low salinity Group A, the fraction of most subgroups of *Bathyarchaeota* and *Thermoprofundales* were positively correlated with ammonium and TOC, while negatively correlated with salinity and depth. Of note, these archaeal subgroups showed clearly opposite correlation patterns in Group B samples, regarding as salinity, ammonium and TOC particularly. Bathy-6, -15, and -17 were negatively correlated with salinity (*p* < 0.05) in low salinity environments while displayed strong positive correlations with salinity (*p* < 0.05) in high salinity conditions. The divergent salinity preference indicated the different composition for bathyarchaeotal subgroups, and the community shift within subgroups along the salinity gradient.

**FIGURE 6:**
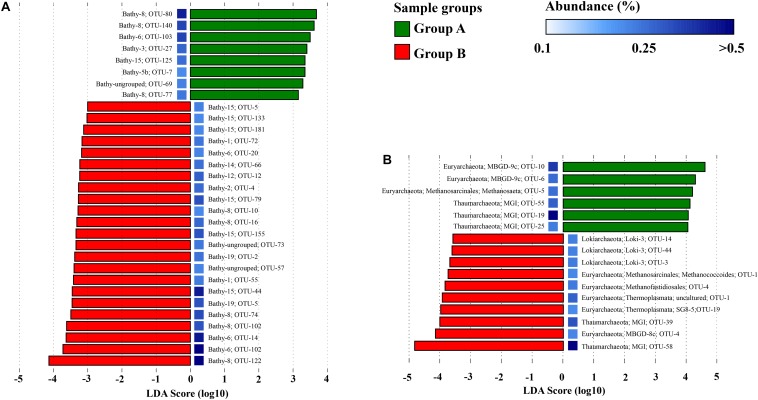
Differential distributions of the subgroups of *Bathyarchaeota* **(A)**, and *Thaumarchaeota*, *Euryarchaeota*, and *Lokiarchaeota* **(B)** in a Group A (low-salinity) and Group B (relatively high salinity) based on logarithmic LDA scores. The color of the squares indicates the abundance of OTUs.

LEfSe analysis was performed to evaluate the distribution of major subgroups of *Bathyarchaeota*, *Thaumarchaeota*, *Euryarchaeota*, and *Lokiarchaeota* on the OTU level in sample groups (Figure 6). For *Bathyarchaeota*, different OTUs showed different salinity preference. However, OTUs within the same subgroup showed different salinity preference; for example, Bathy-6_OTU103 was enriched in low-salinity samples (Group A), while Bathy-6_OTU14 and Bathy-6_OTU102 were more abundant in high-salinity samples (Group B). For MG-I, different OTUs presented different salinity preference, while OTUs from the Loki-3 subgroup only showed high-salinity preference. Within *Thermoprofundales*, MBGD9c_OTU10 and MBGD9c_OTU6 were highly enriched in low-salinity samples (Group A), while MBGD8c_OTU4 was enriched in high-salinity samples (Group B).

**FIGURE 7:**
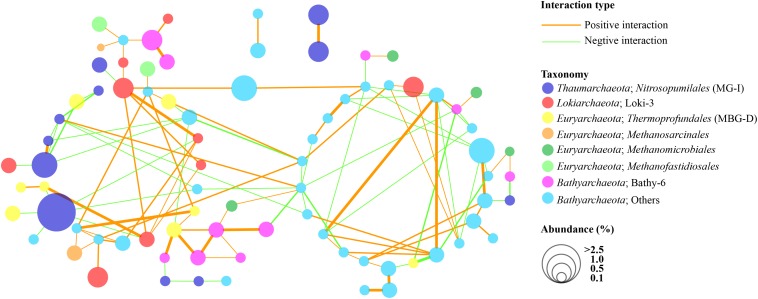
Co-occurrence and co-exclusion networks of archaeal communities in the PRE surface sediments on the OTU level (average abundance fraction > 0.1%). Edge width represents the intensity of interactions. Only interactions with bathyarchaeotal OTUs, and confirmed by at least two out of three analysis methods used (i.e. Pearson, Spearman, and Bray–Curtis) at *p* < 0.01 are presented.

The co-occurrence patterns of bathyarchaeotal subgroups and other major archaeal subgroups are illustrated by an archaeal network analysis (Figure 7). The network consisted of 81 nodes and 119 edges, with 71 edges representing positive interactions and 48 edges representing negative interactions. As shown, OTUs in the bathyarchaeotal subgroups exhibited complex correlations with each other, except for Bathy-6, which showed relatively few interactions with other *Bathyarchaeota* but was positively correlated with some OTUs of Loki-3, *Thermoprofundales*, and *Methanomicrobiales*. In addition, some subgroups of *Bathyarchaeota* also showed positive correlations with *Methanomicrobiales*, *Methanofastidiosales*, and *Methanosarcinales*. However, MG-I showed negative interactions with other archaea, although their relative abundance was high. By contrast, OTUs of Loki-3 showed positive correlations with many *Bathyarchaeota* and *Thermoprofundales*.

## Discussion

### The Highly Diverse Archaeal Communities in the PRE

According to many studies, while the microbial community structure in different environments is unique, it is largely shaped by the local physicochemical parameters (Fierer et al., 2003; Lozupone et al., 2007). However, physicochemical parameters in water columns of the PRE are reported to be more sensitive to seasonal variations than sediments and repeat annually, which are mainly influenced by the river discharge, monsoons and climate changes (Yin, 2002; Dong et al., 2004; Wu et al., 2012). Therefore, the sedimentary microbial community is usually relatively stable compared with the aquatic environments, some similar results could be found in previous publications (Jiang et al., 2011; Zhou et al., 2017). A previous survey of the community structure of sedimentary archaea in the region from the lower Pearl River to the coastal SCS stressed that salinity is the dominating factor affecting archaeal communities in the estuarine-coastal ecosystems (Xie et al., 2014). Although in the current study the diversity index did not show strong correlations with the environmental parameters (Supplementary Table S5), there were significant (*p* < 0.05) differences in terms of community composition on the OTU level in samples from different groups (Supplementary Table S5). These differences were also apparent in the PCoA data (Supplementary Figure S4). We showed that *Bathyarchaeota* dominated in all samples, except for F101 and ZJD, in which *Thaumarchaeota* was the top-ranked fraction (Figure 2A). This was consistent with a previous report that *Bathyarchaeota* dominate in the mid-latitude estuarine sediments, followed by *Thaumarchaeota* and *Euryarchaeota* (Liu et al., 2018).

Ever since members of *Bathyarchaeota* were first detected in a hot spring environment (Barns et al., 1996), their habitat has been shown to range from the land to deep ocean, manifesting an appreciable diversity of this archaeal phylum (Kubo et al., 2012; Zhou et al., 2018a). Generally, Bathy-8 and Bathy-15 are widely detected from marine sediments, while Bathy-6 is the terrestrial subgroup abundant in soil and fresh water sediments (Xiang et al., 2017; Zhou et al., 2018a). We identified 22 subgroups of *Bathyarchaeota*, with Bathy-8 as the dominant subgroup, followed by Bathy-15 and Bathy-6 (Figure 2B). Besides, previous reports suggested that different subgroups dominate in different sedimentary environments. For example, Bathy-6 was found to be dominant in the surface sediments of mangrove wetlands (Pan et al., 2019), in the White Oak River estuary (Lazar et al., 2015), and Lake Cisó (Fillol et al., 2015); Bathy-2 dominated at the surface of the open ocean seafloor (Yu et al., 2017); Bathy-11 was the dominant group in the surface sediment of Lake Vilar (Fillol et al., 2015). The dominance of both terrestrial and marine subgroups in our study indicate a high diversity of *Bathyarchaeota* in estuarine ecosystems.

We observed that the class *Nitrosopumilales* (MG-I) dominated in *Thaumarchaeota* (Supplementary Figure S3C), which was consistent with previous surveys (Liu et al., 2014; Xie et al., 2014). Loki-3, as the most abundant subgroup of *Lokiarchaeota*, usually inhabits the estuarine and marine sediments (Cai et al., 2019), which is consistent with the data on the PRE surface sediments presented herein (Supplementary Figure S3D). *Thermoprofundales* have been previously categorized into 16 subgroups, of which MBGD-8a and -8c are enriched in saline marine sediments; MBGD-9a and -9c are mostly found in non-saline environments, such as the freshwater, soil, and lake sediments; while MBGD-11 and -12 show no preference in terms of salinity, and are found in a variety of habitats (Zhou et al., 2019). Notably, in the current study, MBGD-8c was the dominant subgroup, followed by MBGD-8a, -12, -11, -9c, and -9a (Supplementary Figure S3B), which indicated that the habitats of these major subgroups are broader than previously assumed, which warrants further investigation. The current study adds more genetic evidence on the high diversity of archaea in a subtropical eutrophic estuary, especially for the detailed composition of *Bathyarchaeota* and *Thermoprofundales*.

### The Abundant *Bathyarchaeota* in the PRE

The abundance of archaea and bacteria was reported to be comparable especially in ocean margins (Hoshino and Inagaki, 2018), for example the abundance of archaea and bacteria was about 5–8 × 10^8^ copies/g and 1–5 × 10^9^ copies/g in the surface sediments of Aarhus Bay (Chen et al., 2017); in the surface sediments of Mediterranean, the abundance of archaea was about 0.7–1.0 × 10^8^ copies/g and the abundance of bacteria was about 0.5–2.5 × 10^8^ copies/g (Pala et al., 2018). The abundance of archaea was higher in this study, which might indicate high microbial activities of archaea in this region. Although several studies had surveyed the archaeal composition in the PRE sediments (Liu et al., 2014; Xie et al., 2014; Zhou et al., 2018b), they seldom analyzed the abundance and composition of *Bathyarchaeota* in the region. Our results show the abundance of *Bathyarchaeota* varied from 1.43 × 10^8^ to 1.22 × 10^9^ copies/g d.w. (Figure 3 and Supplementary Table S3). The TOC content is reportedly significantly and positively correlated with the abundance of *Bathyarchaeota* in the SCS along the sediment core depth (Yu et al., 2017). Another study also suggested that the abundance of *Bathyarchaeota* increases along the sediment depth (Liu et al., 2014), but decreases as the reductive redox conditions of the sediment decrease (Lazar et al., 2015). In the current study, however, we did not observe any significant correlations between the physicochemical parameters and *Bathyarchaeota* abundance (Supplementary Table S4). This could be explained by the notion that the estuary is a dynamic ecosystem that is always co-influenced by several physicochemical parameters, including the salinity, pH, temperature, organic content, etc. (Vieira et al., 2007; Webster et al., 2014). Further, the abundance of the bathyarchaeotal 16S rRNA gene in the surface sediments varies with the environment, and was reported to be approximately 3.15 × 10^4^ to 2.18 × 10^5^ copies/g in the SCS (Yu et al., 2017); approximately 3.07 × 10^9^ copies/g in the nearby mangrove (Pan et al., 2019); approximately 3.17 × 10^7^ copies/g to 3.79 × 10^8^ copies/g in the White Oak River (Kubo et al., 2012); and approximately 1.1 × 10^8^ and 1.8 × 10^8^ copies/g in the Lake Cisó and Lake Vilar, respectively (Fillol et al., 2015). Notably, the distribution of bathyarchaeotal subgroups is environment-specific. For example, Bathy-6 prefers the suboxic shallow sediments with low sulfide content, while Bathy-1, -5, and -8 are enriched in deeper sediments under higher reducing conditions (Lazar et al., 2015); and Bathy-15 dominates in organic matter-rich sediments (Fillol et al., 2015). Genomic evidence revealed the degradation capacities of diverse protein, lipid, cellulose, benzoate as well as glycogen and galactan for members of the Bathy-8, -15 and -6 (Lloyd et al., 2013; Lazar et al., 2016; Feng et al., 2019). Besides, members of Bathy-6 was believed to break down extracellular carbohydrates by encoded genes for carbohydrate-active enzymes (CAZys) (Lazar et al., 2016; Maus et al., 2018). The high proportions of Bathy-8, Bathy-15 and Bathy-6 is consistent with previous surveys about the eutrophication caused by massive anthropogenic inputs in the PRE (Huang et al., 2003; Zhou et al., 2004). Therefore, the observed variation of *Bathyarchaeota* abundance is probably associated with the different environmental adaptation capacity of the different subgroups, underscoring a high metabolic diversity for carbon sources of *Bathyarchaeota*.

### Salinity and Ammonium Influenced the Distribution of *Bathyarchaeota*

Similar to a previous study of a farther offshore region of the PRE and SCS (Liu et al., 2014), we here observed a strong effect of salinity on the community structure of *Bathyarchaeota*. A former study suggested that salinity is the best explanatory variable for the distribution of *Bathyarchaeota*, and Bathy-1 and -8 were identified as the marine indicator subgroups, while Bathy-5b and -11 were considered as the freshwater indicators (Fillol et al., 2016). Salinity showed huge influence on the archaeal community in the PRE including *Bathyarchaeota* (Figure 4), and the community composition was significant different between the low-salinity Group A and high-salinity Group B (Supplementary Figure S4 and Supplementary Table S6). Moreover, the archaeal subgroups revealed inverse correlation patterns with physicochemical parameters in low and high salinity samples, especially for *Bathyarchaeota* (Figure 5), implying different adaption strategies for them in different environments. Intriguingly, in the current study, some OTUs of Bathy-8, -6, and -15 showed a preference for low-salinity sediments, while some were enriched in high-salinity samples (Figure 6). As the PRE is a highly dynamic region in which fresh waters mix with saline waters, the different salinity preferences may indicate high diversity within the bathyarchaeotal subgroups in the PRE.

Ammonium, as a crucial substrate in the nitrogen cycle, plays an irreplaceable role in shaping the terrestrial and aquatic microbial communities (Francis et al., 2005; Nicol et al., 2008). In the current study, the ammonium concentration was higher in low-salinity Group A samples located in the upper PRE (Figure 1 and Supplementary Table S2), which are closer to the anthropogenic inputs of ammonium by river discharge, than in other regions. In addition to the observation that ammonium was the best explanatory variable for the distribution of archaea in the current study, it was also strongly positively correlated with some major archaeal subgroups (*p* < 0.05), such as Bathy-6, members of *Thermoprofundales*, and methanogens in the PRE (Figures 4, 5). According to previous studies, ammonium shapes the methanogenic community composition and *Methanosarcinales* could be the dominant methanogens in ammonium-rich environments (Fotidis et al., 2013; Lü et al., 2013), which was consistent with the current study. The high abundance of Bathy-6 were also reported in the surface sediments or soils with high ammonium concentration, like freshwater karstic lakes (Fillol et al., 2015) and mangroves (Pan et al., 2019). Similarly, the strong positive correlation between Bathy-6 and ammonium was also observed, which might indicate potential utilizations of ammonium for this subgroup, and imply the environmental advantages for them in high ammonium conditions. Therefore, in the eutrophic PRE microbial ecosystems, ammonium is probably a key factor, together with salinity, affecting the archaeal community structure.

### Close Interactions Between Bathyarchaeotal Subgroups and Other Archaea

Genomic analysis of *Bathyarchaeota* from diverse environments indicated that these archaea are able to anaerobically degrade detrital proteins, carbon compounds (including carbohydrates, fatty acids, acetate, urea, and aromatic compounds), and methane and methylated compounds (Lloyd et al., 2013; Lloyd, 2015; Meng et al., 2014; Seyler et al., 2014; Evans et al., 2015; Lazar et al., 2016), which emphasizes their vital roles in global carbon cycling. Therefore, the interactions of *Bathyarchaeota* with other microbes should be investigated in detail. Although the primer set employed in this study was targeting at archaea, the interactions between bacteria and Bathyarchaeota are vital to understand the sedimentary nutrient cycling, which should not be ignored and need further exploration. Most members of *Bathyarchaeota* are considered as acetogens and they are capable for gaining energy through the reductive acetyl-CoA [Wood–Ljungdahl (WL)] pathway and fermentation of variety organic substrates (Lloyd et al., 2013; He et al., 2016; Feng et al., 2019). The acetate generated by *Bathyarchaeota* may fuel the heterotrophic microbes and acetoclastic methanogens, also facilitate the carbon transformation in the subsurface sediments (He et al., 2016; Lazar et al., 2016). Besides, members of Bathy-8 were considered as methylotrophic methanogens and may have potential interactions with methanotrophs and the sulfate-reducing bacteria functioned through electron transfer (He et al., 2016; Zhou et al., 2018a). As shown in the current study, within *Bathyarchaeota*, the interactions were very complex for most OTUs from different subgroups, indicating a high diversity and niche differences (Figure 7). We also noted a close co-occurrence of some bathyarchaeotal subgroups and *Lokiarchaeota*, as well as some OTUs of *Thermoprofundales*, *Methanomicrobiales*, *Methanofastidiosales*, and *Methanosarcinales*, which may imply a syntrophic association (He et al., 2016; Xiang et al., 2017; Pan et al., 2019).

Interestingly, Bathy-6 exhibited a co-occurrence trend that was completely different than that of other bathyarchaeotal subgroups. According to a recent study, Bathy-6 are a distinct group in the mangrove sediments, as they are negatively correlated with other bathyarchaeotal subgroups, *Lokiarchaeota*, and *Thermoplasmata* (Pan et al., 2019). However, in the current study, we observed some strong positive interactions of Bathy-6 with OTUs of *Lokiarchaeota*, *Thermoprofundales*, and *Methanomicrobiales*, suggesting the Bathy-6 may have different metabolic capacities in different environments. Previous genomic analysis of Bathy-6 revealed its capacity to uptake and metabolize a wide range of carbohydrates and proteins, including extracellular plant-derived compounds, monosaccharides, and polysaccharides (Lazar et al., 2016; Maus et al., 2018), which might explain the close connection with complex TOC in the estuarine sediments. Further, genomic analysis revealed that the *nrfD* gene (encoding a nitrite reductase) and *narK* gene (encoding a nitrate/nitrite transporter) are encoded in bins of Bathy-6, indicating a possible dissimilatory nitrite reduction to ammonium (DNRA) pathway and a nitrate/nitrite transport system in these organisms (Lazar et al., 2016). In addition, the *narG* and *narY* genes (encoding a nitrate reductase) have been identified in the reconstructed genomes of *Thermoprofundales*, suggesting a potential capacity for denitrification (Zhou et al., 2019). These findings imply the important roles for *Bathyarchaeota* and *Thermoprofundales* in the sedimentary nitrogen cycle, as their potential metabolic capacities in the initial step of denitrification or dissimilatory nitrate reduction to ammonia. Moreover, *Bathyarchaeota* and *Thermoprofundales* were also reported to share similar inferred pathways like acetogenesis and protein degradations (Lazar et al., 2016, 2017), and the strong co-occurrence relationship between them in our study indicate a potential close symbiosis or synergism connection for them.

Conclusively, although genomic data reveals some unique metabolic potentials for *Bathyarchaeota*, the physiological and biochemical characteristics of most *Bathyarchaeota* members are still unknown due to the lack of pure cultures for this archaeal group. The current study observed the dominant *Bathyarchaeota* in nutrient-rich sediments and corroborates the possible syntrophic interactions between them and other archaeal group, which may furtherance the improvement of enrichment and cultivation experiments.

## Data Availability Statement

The datasets generated for this study can be found in the NCBI BioProject accession no. PRJNA574836.

## Author Contributions

DZ and ML conceived the study. JP and ZL determined all the physicochemical parameters. HL and CZ provided the sediment samples. DZ performed laboratory work and analyzed the data and wrote the manuscript with the help from all co-authors.

## Conflict of Interest

The authors declare that the research was conducted in the absence of any commercial or financial relationships that could be construed as a potential conflict of interest.
