# Supplementary material for: The Distribution of Bathyarchaeota in Surface Sediments of the Pearl River Estuary Along Salinity Gradient
Source: Front Microbiol. 2020 Feb 27;11:285. doi: 10.3389/fmicb.2020.00285 (PMC7056671; doi:10.3389/fmicb.2020.00285)
Supplement: Supplementary file 1 [file Presentation_1.pdf]

Supporting Information

The Distribution of Bathyarchaeota in Surface Sediments of the Pearl River Estuary Along Salinity Gradient

Dayu Zou[1,2,3], Jie Pan[3], Zongbao Liu[3], Chuanlun Zhang[4], Hongbin Liu[2]#, Meng Li[1,3]#

[a]SZU-HKUST Joint PhD Program in Marine Environmental Science, Shenzhen University, Shenzhen, China

[b]Department of Ocean Science, The Hong Kong University of Science and Technology, Hong Kong SAR, China

[c] Shenzhen Key Laboratory of Marine Microbiome Engineering, Institute for Advanced Study, Shenzhen University, Shenzhen, China

[d]Shenzhen Key Laboratory of Marine Archaea Geo-Omics, Department of Ocean Science and Engineering, Southern University of Science and Technology, Shenzhen, China

*Correspondence: Hongbin Liu, liuhb@ust.hk; Meng Li, limeng848@szu.edu.cn.

List of figures

Figure S1. Rarefaction curves of the Shannon index (A) and observed OTU number (B) of samples.

Figure S2. Sample clustering results.

Figure S3. Community composition of Euryarchaeota, Thaumarchaeota, Lokiarchaeota and Thermoprofundales (MBG-D).

Figure S4. Principal coordinate analysis (PCoA) on total archaeal community (A) and bathyarchaeotal composition in OUT levels.

List of tables

Table S1. Physicochemical parameters of the PRE sediment sample.

Table S2. Results of normality test and difference test of multiple variables between low-salinity and high-salinity sediments samples.

Table S3. Detailed sequencing information, diversity index and qPCR results of samples.

Table S4. Archaeal community composition fractions.

Table S5. Pearson correlation analysis between physicochemical parameters and variables.

Table S6. The analysis of similarities (ANOSIM) for total archaeal community and Bathyarchaeota composition in OUT level between high and low salinity.

Figures

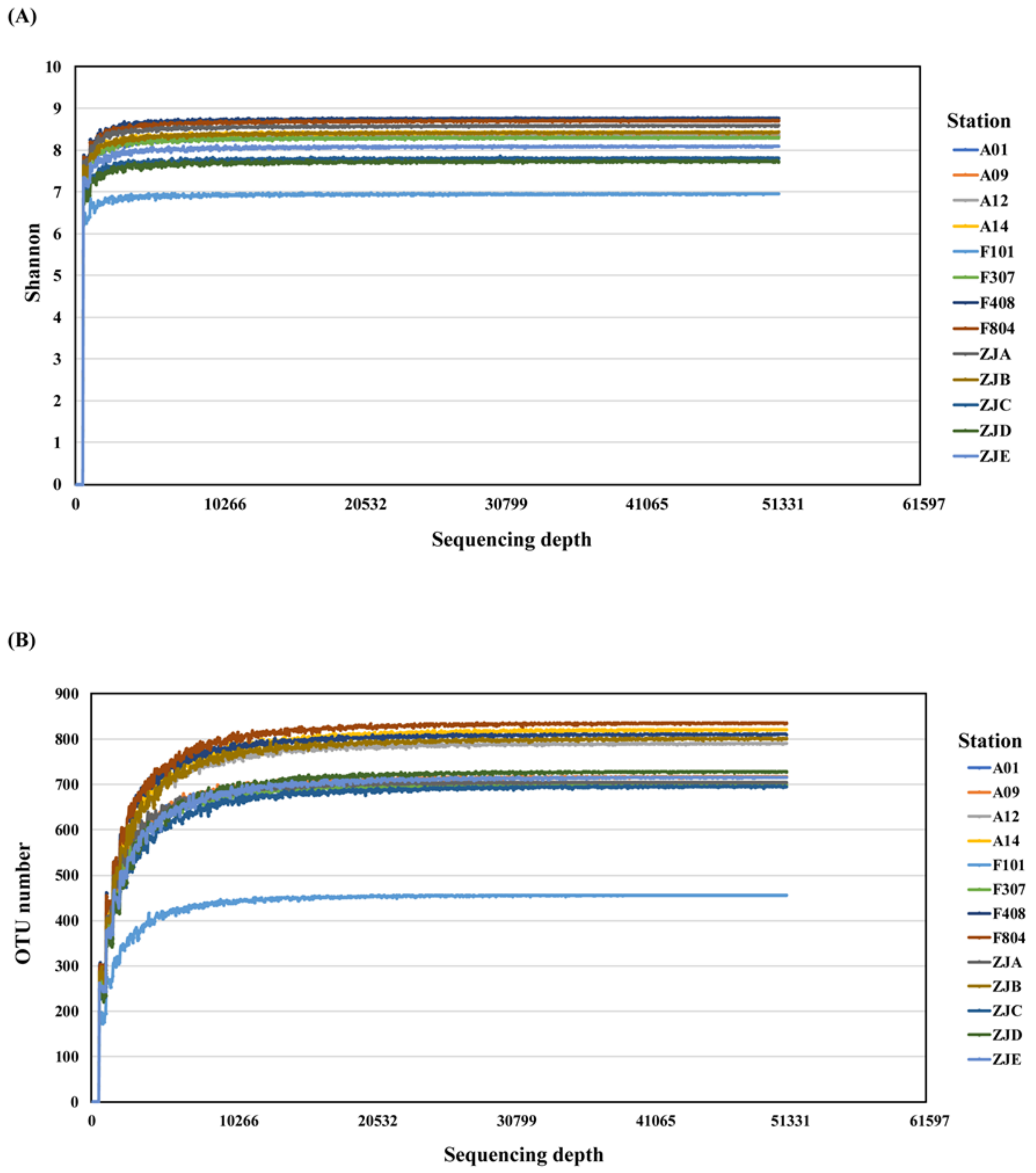

FIG S1 Rarefaction curves of the Shannon index (A) and observed OTU number (B) of samples at different sampling depth.

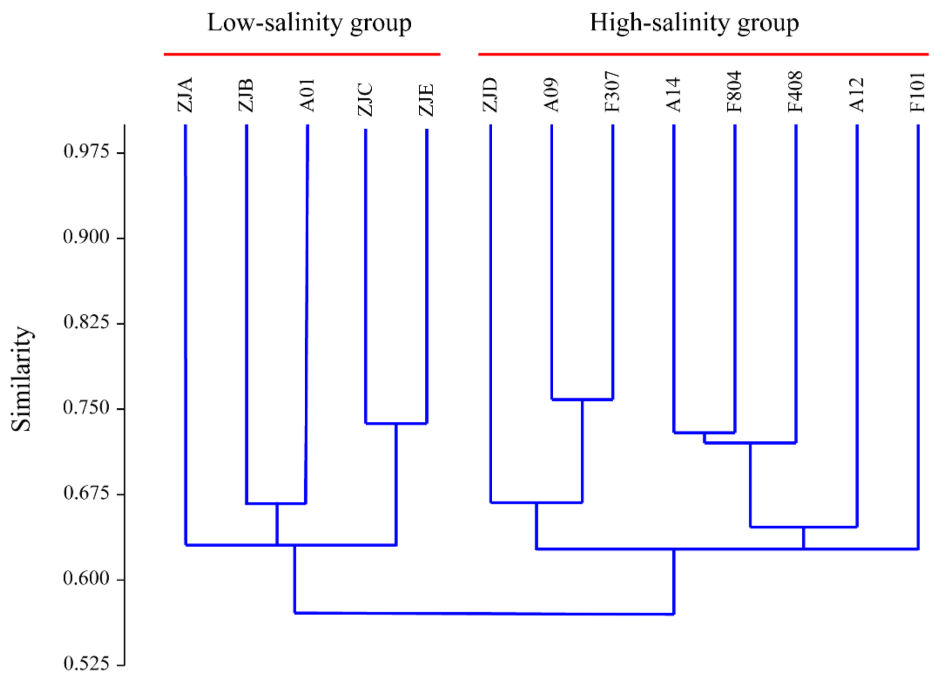

FIG S2 Sample clustering results based on archaeal OTUs

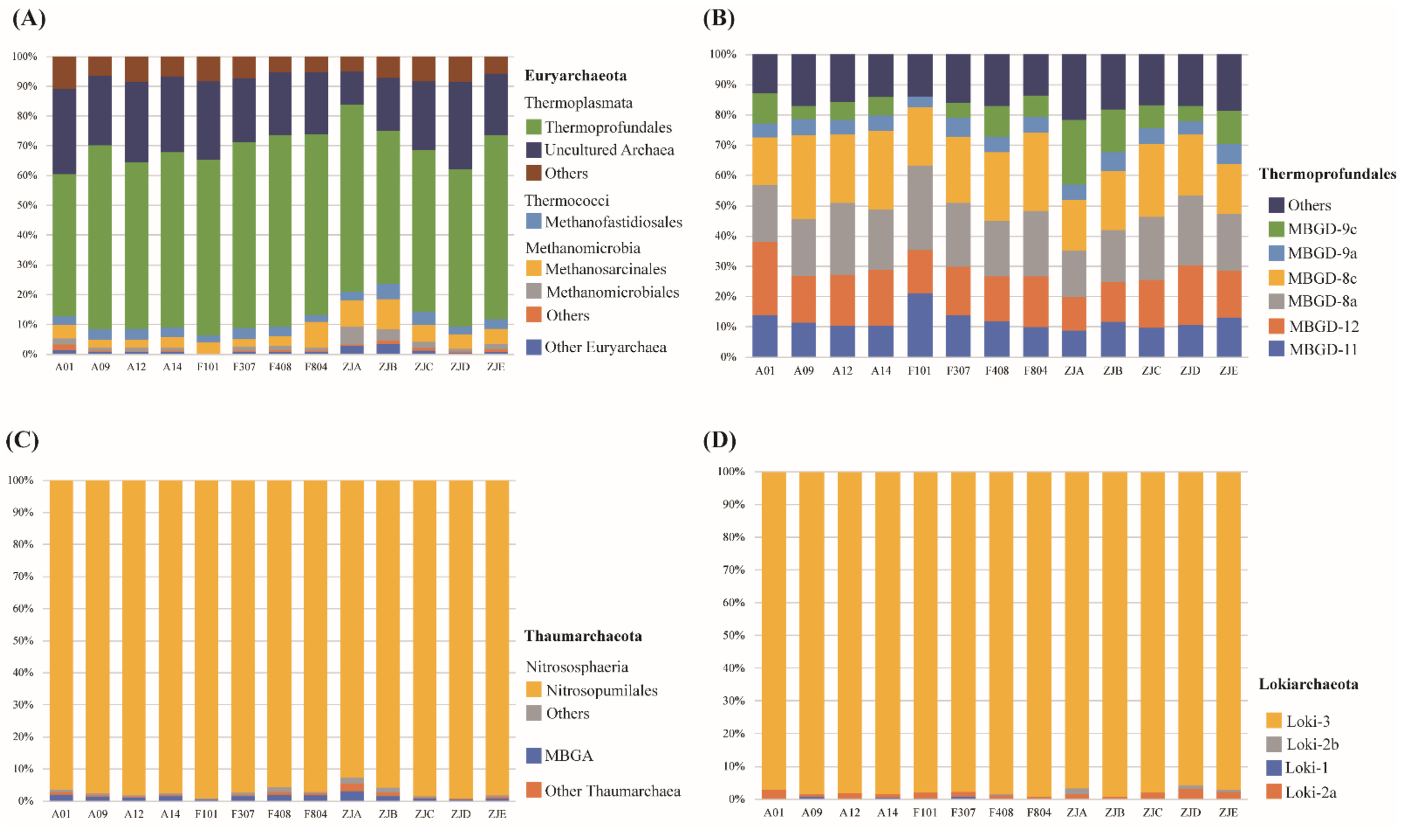

FIG S3 Community composition of Euryarchaeota, Thaumarchaeota, Lokiarchaeota and Thermoprofundales (MBG-D).

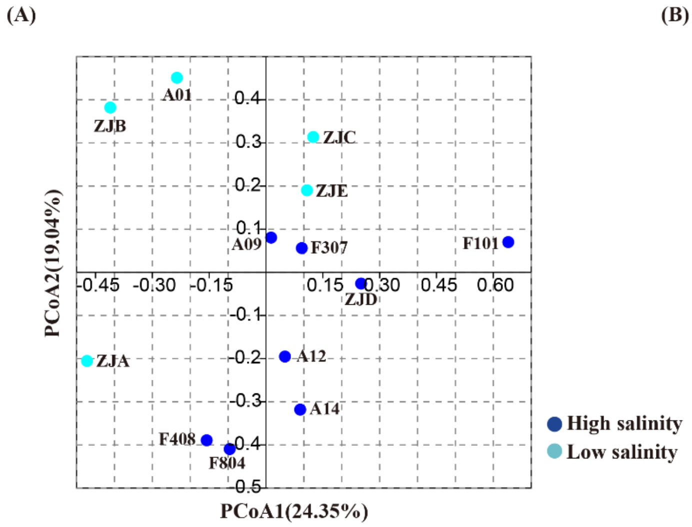

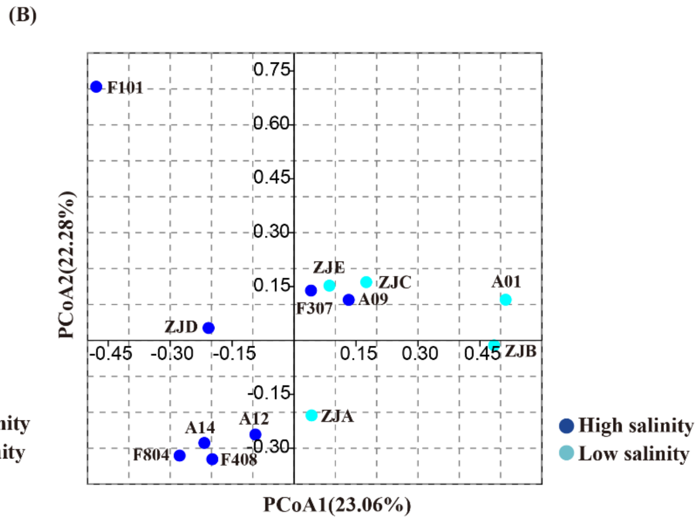

FIG S4 Principal coordinate analysis (PCoA) on total archaeal community (A) and bathyarchaeotal composition in OUT levels.

TABLES

TABLE S1 Physicochemical parameters of the PRE sediment samples

**Station**	Longitude	Latitude	Depth^{+} (m)	Salinity* (‰)	pH*	NH_4^+ (mgN/kg)	NO_2^- (mgN/kg)	NO_3^- (mgN/kg)	TOC (mgC/kg)	TN (mgN/kg)
**A01**	113.65	22.74	9	1.1	7.33	22.55	0.268	1.552	1483	2236
**A09**	113.80	22.21	25	33.4	7.43	11.51	0.270	1.032	1374	1933
**A12**	113.90	21.99	26	34.4	7.67	8.68	0.328	1.690	846.1	1847
**A14**	113.96	21.85	29	34.4	7.70	9.98	0.298	1.758	584.1	1321
**F101**	113.12	21.82	13	33.7	7.30	11.43	0.286	0.842	995.2	1557
**F307**	113.71	21.64	39	34.4	7.43	20.70	0.348	1.724	1671	2590
**F408**	113.88	21.71	41	34.4	7.64	9.60	0.332	1.456	777.7	1607
**F804**	114.36	21.96	41	34.5	7.54	10.29	0.288	1.478	789.7	2085
**ZJA**	113.48	23.07	11	0.3	7.50	107.66	0.234	1.430	2431	3247
**ZJB**	113.72	22.63	15	10.3	7.79	61.96	0.256	1.188	2238	3535
**ZJC**	113.80	22.32	12	20.8	7.69	13.00	0.228	1.704	1054	1046
**ZJD**	113.67	21.97	8	31.3	7.92	23.14	0.374	2.218	1445	2980
**ZJE**	113.74	22.12	10	26.2	7.84	20.43	0.292	1.108	1837	2336

+: Water depth upon the surface sediments.

*: Parameters of pore waters extracted from each sediment.

TABLE S2 Results of normality test and difference test of multiple variables between low-salinity and high-salinity sediments samples (data distribution was tested by Kolmogorov-Smirnov method; if it was normal, T test was applied for the significance test, otherwise, Mann-Whitney test was applied; significant level was set as 0.05)

Variable	Test of normality	Method	Test of difference
Depth	0.415	t-test	0.001
Salinity	0.331	t-test	0.009
pH	0.975	t-test	0.664
NH_4^+	0.036	Mann–Whitney test	0.007
NO_2^-	0.922	t-test	0.200
NO_3^-	0.925	t-test	0.620
TOC	0.863	t-test	0.013
TN	0.993	t-test	0.085
archaea_Simpson	0.210	t-test	0.718
archaea_Shannon	0.286	t-test	0.870
archaea_observed_OTUs	0.173	t-test	0.882

TABLE S3 Clean read numbers, diversity indices, and abundance of the total archaea and *Bathyarchaeota* 16S rRNA genes in samples

	**Sequencing information**	**Diversity index**			**Archaea abundance**	***Bathyarchaeota* abundance**
**Station**	Clean reads[a]	Shannon	Simpson	OTUs[b]	Abundance (gene copies/g)[c]	
**A01**	72,096 (95.56%)	8.3240	0.9916	705	1.4012×10^{9} (1.6293×10^{8})	4.0970×10^{8} (1.1950×10^{7})
**A09**	77,115 (96.37%)	8.3480	0.9917	717	2.6224×10^{9} (2.3868×10^{8})	1.2179×10^{9} (1.6504×10^{7})
**A12**	79,943 (96.87%)	8.4053	0.9912	790	2.1116×10^{9} (4.7360×10^{7})	1.2045×10^{9} (5.3787×10^{7})
**A14**	81,307 (96.46%)	8.4422	0.9903	821	1.2908×10^{9} (3.7816×10^{8})	1.4349×10^{8} (2.7416×10^{6})
**F101**	51,331 (92.70%)	6.9576	0.9692	455	8.0600×10^{8} (4.8640×10^{7})	1.7963×10^{8} (1.2382×10^{6})
**F307**	73,793 (95.88%)	8.3110	0.9909	698	2.2284×10^{9} (1.1120×10^{8})	5.4235×10^{8} (2.3836×10^{7})
**F408**	74,675 (96.72%)	8.7765	0.9949	811	9.8120×10^{8} (2.7934×10^{8})	2.3130×10^{8} (1.1196×10^{7})
**F804**	74,772 (96.94%)	8.7196	0.9941	835	1.6136×10^{9} (2.3700×10^{8})	2.4680×10^{8} (1.4449×10^{7})
**ZJA**	66,856 (97.65%)	8.5957	0.9948	705	1.6508×10^{9} (1.3340×10^{8})	1.5122×10^{8} (2.2679×10^{6})
**ZJB**	76,835	8.4336	0.9897	801	1.5260×10^{9}	3.8698×10^{8}

	(96.33%)				(2.9933×10^7)	(2.4235×10^7)
**ZJC**	78,675<br>(97.10%)	7.8192	0.9820	696	1.0832×10^9<br>(1.4613×10^8)	1.9760×10^8<br>(4.5121×10^6)
**ZJD**	78,009<br>(95.87%)	7.7440	0.9780	728	3.6604×10^9<br>(3.8903×10^7)	3.4798×10^8<br>(9.1444×10^6)
**ZJE**	78,283<br>(95.69%)	8.1019	0.9867	716	1.9304×10^9<br>(2.4120×10^8)	3.3182×10^8<br>(1.9627×10^7)

a: The number of archaeal reads and its proportion (in brackets) in total clean reads.

b: The number of archaeal OTUs for each sample.

c: The number and error (in brackets) of bathyarchaeotal 16S rRNA gene copies per gram dry sediments for each sample.

TABLE S4 Archaeal community composition fractions

Taxa	A01	A09	A12	A14	F101	F307	F408	F804	ZJA	ZJB	ZJC	ZJD	ZJE
**Total archaea**													
Bathyarchaeota	0.5959	0.5798	0.5927	0.5184	0.3613	0.5326	0.6091	0.5570	0.5699	0.6549	0.4691	0.3463	0.5138
Crenarchaeota	0.0058	0.0035	0.0076	0.0048	0.0011	0.0041	0.0057	0.0040	0.0331	0.0066	0.0037	0.0033	0.0040
Euryarchaeota	0.1195	0.1239	0.0998	0.1193	0.1078	0.1451	0.1321	0.1591	0.1990	0.1139	0.0725	0.1354	0.0963
Hydrothermarchaeota	0.0237	0.0066	0.0120	0.0118	0.0242	0.0103	0.0073	0.0099	0.0055	0.0053	0.0143	0.0155	0.0087
Lokiarchaeota	0.0411	0.0590	0.0529	0.0700	0.0746	0.0625	0.0690	0.0671	0.0409	0.0427	0.0522	0.0580	0.0531
Odinarchaeota	0.0062	0.0099	0.0074	0.0106	0.0100	0.0103	0.0097	0.0086	0.0088	0.0081	0.0124	0.0159	0.0070
Thaumarchaeota	0.1970	0.2059	0.2190	0.2534	0.4106	0.2235	0.1548	0.1827	0.1356	0.1603	0.3663	0.4136	0.3090
Others	0.0109	0.0113	0.0087	0.0118	0.0104	0.0117	0.0123	0.0117	0.0071	0.0082	0.0094	0.0119	0.0079
**Bathyarchaeota**													
Bathy-1	0.0166	0.0227	0.0215	0.0178	0.0111	0.0200	0.0178	0.0214	0.0113	0.0186	0.0200	0.0103	0.0155
Bathy-10	0.0056	0.0130	0.0081	0.0079	0.0047	0.0101	0.0084	0.0120	0.0051	0.0098	0.0048	0.0043	0.0058
Bathy-11	0.0090	0.0041	0.0035	0.0034	0.0005	0.0057	0.0190	0.0081	0.0155	0.0103	0.0044	0.0030	0.0108
Bathy-12	0.0496	0.0480	0.0642	0.0256	0.0173	0.0358	0.0285	0.0241	0.0175	0.0456	0.0318	0.0188	0.0334
Bathy-13	0.0205	0.0196	0.0280	0.0271	0.0105	0.0205	0.0286	0.0302	0.0163	0.0233	0.0181	0.0170	0.0184
Bathy-14	0.0106	0.0124	0.0188	0.0156	0.0056	0.0124	0.0179	0.0209	0.0097	0.0113	0.0100	0.0091	0.0090
Bathy-15	0.0961	0.1065	0.0865	0.1135	0.0840	0.0934	0.1413	0.1116	0.1167	0.0978	0.0798	0.0745	0.0974
Bathy-16	0.0044	0.0023	0.0017	0.0016	0.0018	0.0020	0.0017	0.0016	0.0015	0.0017	0.0042	0.0019	0.0024
Bathy-17	0.0468	0.0555	0.0523	0.0723	0.0441	0.0593	0.0740	0.0733	0.0564	0.0553	0.0456	0.0404	0.0518
Bathy-18	0.0003	0.0006	0.0004	0.0006	0	0.0004	0.0004	0.0005	0.0010	0.0004	0.0004	0.0004	0.0004
Bathy-19	0.0068	0.0058	0.0170	0.0042	0.0050	0.0040	0.0045	0.0038	0.0023	0.0042	0.0035	0.0033	0.0042
Bathy-2	0.0092	0.0077	0.0095	0.0064	0.0033	0.0072	0.0063	0.0086	0.0034	0.0076	0.0043	0.0038	0.0049
Bathy-20	0.0010	0.0015	0.0022	0.0024	0.0015	0.0020	0.0029	0.0031	0.0013	0.0011	0.0017	0.0011	0.0017
Bathy-22	0	0	0.0001	0	0	0	0	0	0	0	0	0	0
Bathy-3	0.0325	0.0219	0.0441	0.0222	0.0127	0.0186	0.0221	0.0223	0.0144	0.0271	0.0175	0.0130	0.0197
Bathy-4	0.0154	0.0110	0.0212	0.0119	0.0053	0.0086	0.0112	0.0128	0.0055	0.0100	0.0091	0.0082	0.0102
Bathy-5a	0	0	0	0	0	0.0003	0	0	0	0	0	0	0
Bathy-5b	0.0022	0.0021	0.0022	0.0023	0.0002	0.0021	0.0041	0.0019	0.0115	0.0031	0.0018	0.0017	0.0029
Bathy-5bb	0.0047	0.0040	0.0034	0.0044	0.0013	0.0043	0.0107	0.0076	0.0200	0.0065	0.0027	0.0032	0.0076

Bathy-6	0.0678	0.0669	0.0455	0.0596	0.0446	0.0762	0.0833	0.0620	0.1635	0.0949	0.0661	0.0474	0.0760
Bathy-8	0.1792	0.1559	0.1594	0.1045	0.0996	0.1338	0.1054	0.1139	0.0755	0.2073	0.1289	0.0746	0.1269
Bathy-9	0.0025	0.0035	0.0029	0.0015	0.0027	0.0025	0.0023	0.0016	0.0012	0.0033	0.0032	0.0013	0.0027
Ungrouped	0.0149	0.0149	0.0153	0.0139	0.0055	0.0135	0.0186	0.0156	0.0202	0.0157	0.0113	0.0092	0.0120
**Euryarchaeota**													
Methanomicrobiales	0.0026	0.0016	0.0012	0.0016	0	0.0020	0.0020	0.0020	0.0121	0.0040	0.0015	0.0011	0.0018
Methanosarcinales	0.0056	0.0031	0.0026	0.0040	0.0038	0.0036	0.0043	0.0134	0.0172	0.0116	0.0042	0.0066	0.0048
Methanofastidiosales	0.0031	0.0043	0.0037	0.0038	0.0026	0.0052	0.0040	0.0036	0.0058	0.0062	0.0031	0.0037	0.0031
Thermoprofundales	0.0573	0.0765	0.0557	0.0703	0.0637	0.0907	0.0853	0.0968	0.1251	0.0583	0.0395	0.0714	0.0597
Thermoprofundales													
MBGD10	0.0015	0.0043	0.0015	0.0018	0.0023	0.0038	0.0023	0.0025	0.0022	0.0029	0.0023	0.0018	0.0021
MBGD11	0.0079	0.0087	0.0059	0.0074	0.0135	0.0125	0.0101	0.0096	0.0109	0.0068	0.0038	0.0077	0.0079
MBGD12	0.0140	0.0119	0.0093	0.0130	0.0092	0.0145	0.0126	0.0163	0.0139	0.0076	0.0062	0.0140	0.0092
MBGD1	0.0016	0.0021	0.0020	0.0024	0.0008	0.0023	0.0037	0.0036	0.0086	0.0019	0.0011	0.0025	0.0024
MBGD2	0.0007	0.0004	0.0004	0.0003	0	0.0003	0.0009	0.0009	0.0041	0.0015	0.0003	0.0005	0.0003
MBGD3	0.0014	0.0031	0.0016	0.0021	0.0029	0.0046	0.0022	0.0019	0.0026	0.0012	0.0014	0.0029	0.0028
MBGD5	0.0014	0.0017	0.0018	0.0022	0.0027	0.0025	0.0018	0.0021	0.0036	0.0023	0.0011	0.0024	0.0016
MBGD6	0	0	0.0005	0.0001	0.0001	0	0.0005	0.0006	0.0008	0	0.0002	0.0009	0.0002
MBGD8a	0.0106	0.0144	0.0133	0.0139	0.0178	0.0192	0.0159	0.0208	0.0193	0.0101	0.0083	0.0164	0.0112
MBGD8b	0.0006	0.0008	0.0002	0.0002	0	0	0.0003	0.0005	0.0011	0	0.0002	0.0006	0.0004
MBGD8c	0.0091	0.0211	0.0125	0.0182	0.0121	0.0198	0.0191	0.0253	0.0210	0.0113	0.0095	0.0145	0.0099
MBGD9a	0.0027	0.0040	0.0027	0.0035	0.0024	0.0057	0.0045	0.0048	0.0062	0.0036	0.0021	0.0030	0.0039
MBGD9b	0.0002	0.0006	0.0007	0.0008	0	0.0012	0.0026	0.0010	0.0040	0.0008	0	0.0005	0.0013
MBGD9c	0.0056	0.0033	0.0033	0.0043	0	0.0043	0.0087	0.0069	0.0268	0.0083	0.0029	0.0037	0.0065
**Thaumarchaeota**													
Nitrosopumilales	0.1898	0.2011	0.2150	0.2473	0.4072	0.2176	0.1478	0.1773	0.1256	0.1536	0.3605	0.4097	0.3032
**Lokiarchaeota**													
Loki-3	0.0399	0.0581	0.0520	0.0689	0.0731	0.0611	0.0680	0.0666	0.0396	0.0425	0.0512	0.0555	0.0516

TABLE S5 Pearson correlation analysis between physicochemical parameters and both archaeal and bathyarchaeotal 16S rRNA gene abundance, archaeal alpha diversity indices together with abundance of archaeal phyla.

	**Salinity**	**Depth**	**pH**	**NH_4^+**	**NO_2^-**	**NO_3^-**	**TOC**	**TN**
**16S rRNA gene abundance (log10)**								
Archaea	0.1125	-0.0692	0.3416	0.0770	0.4439	0.4085	0.3236	0.5128
Bathyarchaeota	0.1669	0.1092	-0.0845	-0.2666	0.2678	-0.0259	0.0551	0.1275
Bathy%[a]	0.1274	0.1421	-0.2933	-0.3204	0.0524	-0.2179	-0.1680	-0.1757
**Diversity indices**								
Observed OTUs	0.0957	0.4684	0.5098	0.0003	0.1414	0.4289	-0.1218	0.1253
Shannon	-0.0928	0.5481	0.1117	0.2259	-0.0287	0.1798	0.0720	0.2108
Simpson	-0.1483	0.5117	-0.0269	0.2437	-0.1031	0.1024	0.1257	0.1903
**Relative abundance of total archaeal phyla**								
Bathyarchaeota	-0.2857	0.3635	-0.0919	0.2565	-0.2977	-0.2050	0.2105	0.2061
Crenarchaeota	-0.6693*	-0.2032	-0.0931	0.8813***	-0.3891	0.0149	0.5644*	0.4620
Euryarchaeota	-0.2402	0.2484	-0.2511	0.6059*	0.0556	0.1081	0.3584	0.5378
Hydrothermarchaeota	0.0417	-0.3711	-0.3998	-0.3842	0.0766	0.0634	-0.3340	-0.3520
Lokiarchaeota	0.8739***	0.5809*	-0.1336	-0.6484*	0.4663	-0.0679	-0.7322**	-0.5777*
Odinarchaeota	0.3038	-0.0825	0.3525	-0.1455	0.3683	0.5471	-0.1832	-0.0769
Thaumarchaeota	0.2825	-0.4639	0.2134	-0.4017	0.2198	0.1432	-0.2427	-0.3129

a: The proportions of bathyarchaeotal 16S genes abundance on total archaeal 16S genes abundance.

*Significantly correlated at 0.05 level

** Significantly correlated at 0.01 level

*** Significantly correlated at 0.001 level

TABLE S6 The analysis of similarities (ANOSIM) for total archaeal community and Bathyarchaeota composition in OUT level between high and low salinity.

Community	R	p(same)
**Total archaea**	0.3118	0.0102
**Bathyarchaeota**	0.2724	0.0243
